# Proximal visceral endoderm and extraembryonic ectoderm regulate the formation of primordial germ cell precursors

**DOI:** 10.1186/1471-213X-7-140

**Published:** 2007-12-20

**Authors:** Susana M Chuva de Sousa Lopes, Katsuhiko Hayashi, M Azim Surani

**Affiliations:** 1 The Wellcome Trust/Cancer Research UK Gurdon Institute, University of Cambridge, Tennis Court Road, Cambridge CB2 1QN, UK; 2 Hubrecht Institute, Developmental Biology and Stem Cell Research, Uppsalalaan 8, 3584 CT Utrecht, The Netherlands

## Abstract

**Background:**

The extraembryonic tissues, visceral endoderm (VE) and extraembryonic ectoderm (ExE) are known to be important for the induction of primordial germ cells (PGCs) in mice via activation of the bone morphogenetic protein (BMP) signalling pathway. We investigated whether the VE and ExE have a direct role in the specification of PGCs, or in an earlier event, namely the induction of the PGC precursors in the proximal posterior epiblast cells.

**Results:**

We cultured embryonic day (E) 5.75 to E7.0 mouse embryos in an explant-assay with or without extraembryonic tissues. The reconstituted pieces of embryonic and extraembryonic tissues were assessed for the formation of both PGC precursors and specified PGCs. For this, *Blimp1:gfp *and *Stella:gfp *transgenic mouse lines were used to distinguish between PGC precursors and specified PGC, respectively. We observed that the VE regulates formation of an appropriate number of PGC precursors between E6.25–E7.25, but it is not essential for the subsequent specification of PGCs from the precursor cells. Furthermore, we show that the ExE has a different role from that of the VE, which is to restrict localization of PGC precursors to the posterior part of the embryo.

**Conclusion:**

We show that the VE and ExE have distinct roles in the induction of PGC precursors, namely the formation of a normal number of PGC precursors, and their appropriate localization during early development. However, these tissues do not have a direct role during the final stages of specification of the founder population of PGCs.

## Background

In the postimplantation mammalian embryo, germ cells are the first embryonic lineage to become fate-restricted. This was thought to occur during gastrulation at embryonic day 7.25 (E7.25) in the mouse, when a population of about 40 founder primordial germ cells (PGCs) present at the base of allantois are detected. These PGCs exhibit expression of several genes including *Dppa3 *(or *Stella *or *PGC7*) and *Akp2 *(or tissue non-specific alkaline phosphatase, TNAP) (reviewed by [1]). This view has recently been challenged by the unexpected observation of a distinct PGC precursor population present in the embryo already at E6.25–E7.25 [2]. The PGC precursors express *Prdm1 *(or *Blimp1*) and are present in the proximal-posterior epiblast as a row of cells adjacent to the extraembryonic ectoderm. The population of PGC precursors increases from about 6 cells at E6.25 to about 40 cells by the time of specification around E7.25. The initial 6 Blimp1-positive cells may be derived from epiblast cells already present in the posterior part of the embryo; the rest probably derives from epiblast cells scattered throughout the proximal epiblast, but moving to the posterior part of the embryo during gastrulation [3,4].

The visceral endoderm (VE) and the extraembryonic ectoderm (ExE) play an important role in the emergence of PGCs. This was demonstrated by the observation that epiblast cells isolated from E6.0 embryos failed to develop any specified TNAP-positive PGCs, unless they were cultured either on feeder STO cells, or in the presence of both VE and ExE [5-7]. The discovery of the existence of a population of PGC precursors compelled us to investigate whether the VE and ExE were directly involved in the specification of PGCs. For this purpose, we used two recently generated transgenic mouse lines, *Blimp1:gfp *and *Stella:gfp*, which allowed us to distinguish between PGC precursors (Blimp1-positive) and specified PGCs (Blimp1-positive and Stella-positive) [2,8]. We demonstrate that the proximal VE and ExE contribute respectively to the generation of the appropriate number of PGC precursors in their correct location between E6.25–E7.25. More importantly, proximal VE and ExE were not only necessary for the induction of PGC precursors, but also sufficient to induce PGC fate in distal epiblast cells.

## Results

### The VE is required for the formation of Blimp1-positive PGC precursor cells

**Figure 1:**
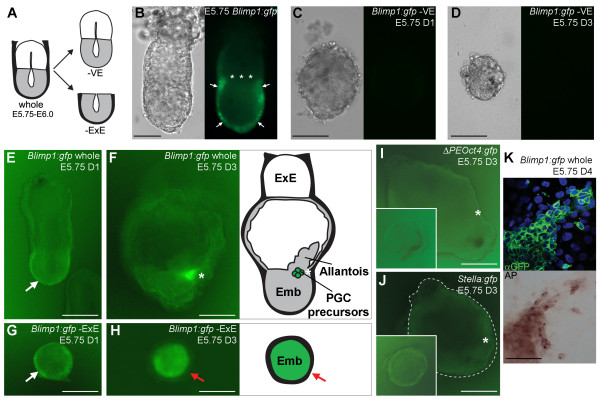
**VE and ExE are both important for the formation of PGC precursors**. (A) Schematic of the experimental design: E5.75 embryos were isolated and either cultured whole or after the surgical removal the VE (-VE) or the ExE (-ExE). (B) E5.75 *Blimp1:gfp *embryo showing GFP signal in the VE (white arrows). PGC precursors emerge approximately 12 hours later in the proximal epiblast region adjacent to the asterisks. (C, D) Explants derived from E5.75 -VE *Blimp1:gfp *embryos cultured for 1 day, D1 (C) and 3 days, D3 (D). (E-H) Explants derived from E5.75 whole and -ExE *Blimp1:gfp *embryos cultured for 1 day, D1 (E, G) and 3 days, D3 (F, H). E, G, white arrows point to Blimp1(GFP)-positive VE; F, white asterisk marks Blimp1(GFP)-positive PGC precursors in the proximal-posterior part of explant; H, red arrow points to Blimp1(GFP)-negative VE, but Blimp1(GFP)-positive epiblast. Cartoons adjacent to the explants elucidate the morphology of the explant. Abbreviations: Emb, tissue derived from epiblast; ExE, extraembryonic ectoderm; thick black line, endoderm. (I, J) Explants derived from E5.75 whole and -ExE *ΔPEOct4:gfp *(I) and *Stella:gfp *(J) embryos cultured for 3 days, D3. White asterisk marks the proximal-posterior part of explant; -ExE explants were inserted in the lower left corner of I and J. K, After 4 days of culture, the cluster of Blimp1(GFP)-positive cells present in explants from whole embryos turns positive for alkaline phosphatase-activity (AP). Scale bar in B-J is 100 μm and K is 25 μm.

To clarify how the VE contributed to the development of PGCs, we assessed their formation in explanted *Blimp1:gfp *E5.75–E6.0 embryos, from which the VE (-VE) was surgically removed (Fig. 1A). In this transgenic model, membrane-tagged GFP is driven by the Blimp1 promoter [2]. At E5.75–E6.0, Blimp1(GFP)-positive PGC precursor cells have not yet emerged in the proximal epiblast (asterisks in Fig. 1B), although Blimp1(GFP) is expressed in the VE (white arrows in Fig. 1B). The expression of the transgenic explants was monitored after 1 and 3 days of culture. In -VE explants, no Blimp1(GFP)-positive cells (n = 0/9) were observed after 1 day (Fig. 1C) or 3 days of culture (Fig. 1D). In whole embryo explants, Blimp1(GFP)-positive cells were still observed in the VE after 1 day of culture (white arrow in Fig. 1E), but after 3 days of culture a clear cluster of Blimp1(GFP)-positive PGC precursors was observed at the boundary with the extraembryonic region (n = 4/5) and Blimp1(GFP) expression was downregulated in the VE (Fig. 1F). Together, these observations suggested an important role of the VE in the induction of the Blimp1-positive PGC precursors. Note that the small size of the -VE explants after 3 days of culture should not hamper the specification of PGCs (Fig. 3).

**Figure 2:**
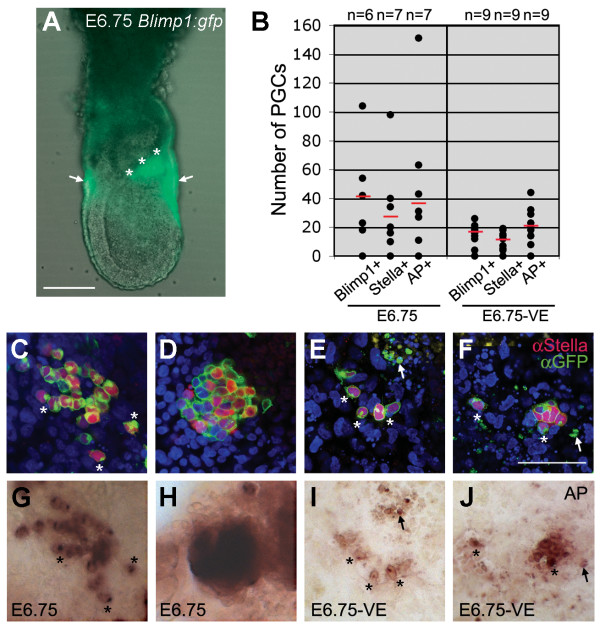
**During gastrulation, the VE is important for the formation of normal number of PGC precursors**. (A) E6.75 *Blimp1:gfp *embryo showing GFP signal in the VE (white arrows) and in the cluster of PGC precursors (adjacent to asterisks). (B) After 3 days of culture, the total number of Blimp1(GFP)-positive, Stella-positive and TNAP-positive cells were analysed in each explant derived from E6.75 whole and -VE embryos. Red bars depict the median, n is the number of individual explants analysed. (C-J) Explants derived from E6.75 whole (C, D, G, H) and -VE (E, F, I, J) immunostained to detect Blimp1(GFP) and Stella (C-F), followed by staining to detect alkaline phosphatase-activity (G-J). Asterisks depict PGCs, arrows depict small cells that are (Blimp1)GFP- and TNAP-positive, but Stella negative. Scale bar in A is 100 μm and in C-J is 50 μm.

**Figure 3:**
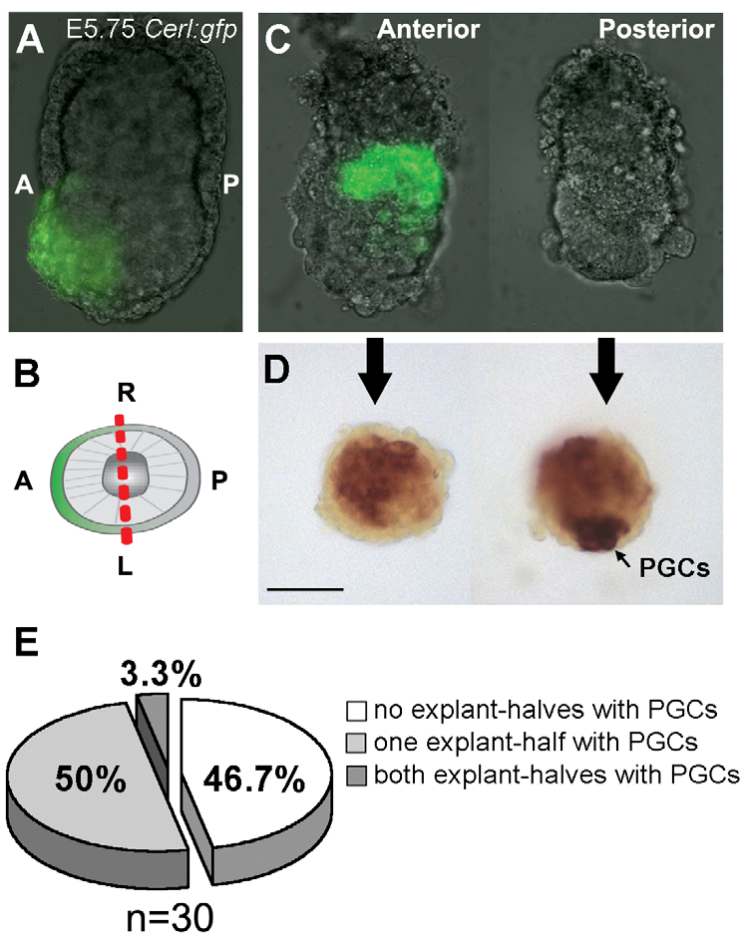
**Only epiblast cells that contact the posterior part of the embryo become PGCs**. (A) E5.75 *Cerl:gfp *embryo showing GFP expression exclusively in the AVE. (B) Cartoon depicting a transverse proximal section of a E5.75 *Cerl:gfp *embryo showing the anterior-posterior (A-P) and left-right (L-R) axes. (C) E5.75 *Cerl:gfp *embryo cut longitudinally through the L-R axis (red-dashed line in B) showing GFP expression in the AVE. (D) E5.75 embryo cut longitudinally through the L-R axis after 4 days culture stained for alkaline phosphatase-activity. Black arrow in D points to PGCs. Scale bar is 100 μm. (E) Quantification of the number or explant-pairs cut longitudinally through the L-R axis that exhibited PGCs after 4 days of culture in one explant-half (posterior), both explants-halves or in none of the explant-halves.

### The ExE prevents ectopic induction of Blimp1-positive PGC precursors

We also studied whether the ExE was involved in formation of PGC precursors or in the subsequent commitment of PGC precursors to PGCs. For this we examined the number of PGC (and PGC precursors) in E5.75–E6.0 *Blimp1:gfp*, *Stella:gfp *and *ΔPEOct4:gfp *explanted embryos cultured in the absence of ExE (Fig. 1A,G–J). After 1 day of culture, similar to whole *Blimp1:gfp *embryo explants, Blimp1(GFP)-positive cells were observed only in the VE of -ExE explants (white arrow in Fig. 1E,G). However, after 3 days of culture of -ExE explants (n = 13/16), Blimp1(GFP) was also downregulated in the VE, but the epiblast-derivative was entirely Blimp1(GFP)-positive (red arrow in Fig. 1H). Note that the dimensions of the epiblast-derivative in both whole and -ExE explants are similar (cartoon in Fig. 1F,H).

When *Stella:gfp *or *ΔPEOct4:gfp *E5.75–E6.0 embryos were cultured for 3 days, no GFP expression (indicative of specified PGCs [8,9]) was observed in whole (Fig. 1I,J) and -ExE explants (bottom left inserts in Fig. 1I,J), suggesting that specification of PGCs had not yet occurred *in vitro*. After 4 days of culture, the Blimp1(GFP)-positive cluster in the explanted whole embryos gave rise to TNAP-positive PGCs (Fig. 1K), whereas the Blimp1(GFP)-expressing cells in -ExE explants remained TNAP-negative (data not shown). This suggested that the Blimp1(GFP)-positive cells that populated the epiblast in the -ExE explants are either no genuine PGC precursors, or if they are PGC precursors, they may need additional signals to commit to the PGC lineage. In any case, these experiments demonstrate that the ExE is (directly or indirectly) important for the restriction of Blimp1(GFP) induction in the epiblast by the VE.

### VE is necessary for the formation of PGC precursors but not for the subsequent PGC-specification event

The absence of Blimp1(GFP)-positive PGC precursor cells in E5.75–E6.0 *Blimp1:gfp *-VE explants could be a secondary effect due to defects in the initiation of morphogenetic movements associated with gastrulation. Therefore, we cultured slightly older gastrulating embryos (E6.75–E7.0), which have a distinct Blimp1(GFP)-positive cluster of ±20 PGC precursors (Fig. 2A and [2]) after having removed the still Blimp1(GFP)-positive VE (-VE). After 3 days in culture, the number of PGCs (Stella-positive and TNAP-positive) observed in *Blimp1:gfp *E6.75–E7.0 -VE explants was reduced compared to explants from whole embryos, but more importantly, the number of Blimp1(GFP)-expressing cells was also correspondingly reduced by half (±40 cells in the whole embryo explant compared to ±20 cells in the -VE explant) (Fig. 2B–J). Note that some of the Blimp1(GFP)-positive cells were small and probably entering apoptosis (contained fragmented DAPI-positive nucleus). These smaller cells failed to upregulate Stella, but were positive for TNAP (including a TNAP-positive cytoplasmic spot) (Fig. 2B,E,F,I,J). In agreement, we observed higher levels of apoptosis using the TUNEL-assay in the -VE explants when compared to whole explants (data not shown).

Notably, the PGC precursors present in the -VE explants undergo specification to PGCs, even though the PGC precursor cluster was rather reduced in size (Fig. 2B–J), indicating that the size of the cluster is not important for specification. Also unexpected was the fact that some of those PGCs acquired a migratory phenotype and were moving out of the PGC-cluster, similarly to PGCs generated from whole E6.5 embryo-explants (asterisks in Fig. 2C,F) and therefore do not seem to require signals from the VE to do so.

### Epiblast cells need to be posterior to become PGCs

The ectopic expression of Blimp(GFP) in the -ExE explants derived from E5.75–E6.0 *Blimp1:gfp *embryos raised the possibility that the ExE may be critical for regionalizing the embryo at E5.75–E6.0, so that the Blimp1-positive PGC precursors can only emerge posteriorly. To investigate this in more detail, we cut E5.75–E6.0 transgenic *Cerl:gfp *embryos [10] longitudinally though the long left-right (L-R) axis (Fig. 3A,B). *Cerberus-like *(*Cerl*) is an anterior visceral endoderm (AVE) marker [11] and therefore in *Cerl:gfp *transgenic embryos GFP expression is present exclusively in the anterior region, allowing clear identification of the embryonic axes (Fig. 3B,C).

The two embryo-halves, each containing epiblast, VE and ExE, were cultured separately for 4 days. After culture, both embryo halves were stained for alkaline phosphatase-activity to assess the presence of TNAP-positive specified PGCs. Notably, the explants-pairs that contained PGCs only exhibited them in one of the explant-halves (Fig. 3D,E) invariably the posterior part (Cerl(GFP)-negative). Only one explant-pair (n = 1/30) showed PGCs in both explant-halves (Fig. 3E). Our data indicates that the anterior VE and anterior ExE were not able to support the formation of PGCs and may even play an active role in repressing Blimp1 expression.

### Proximal VE and ExE are sufficient to induce PGC formation de novo in distal epiblast cells at E6.75

**Figure 4:**
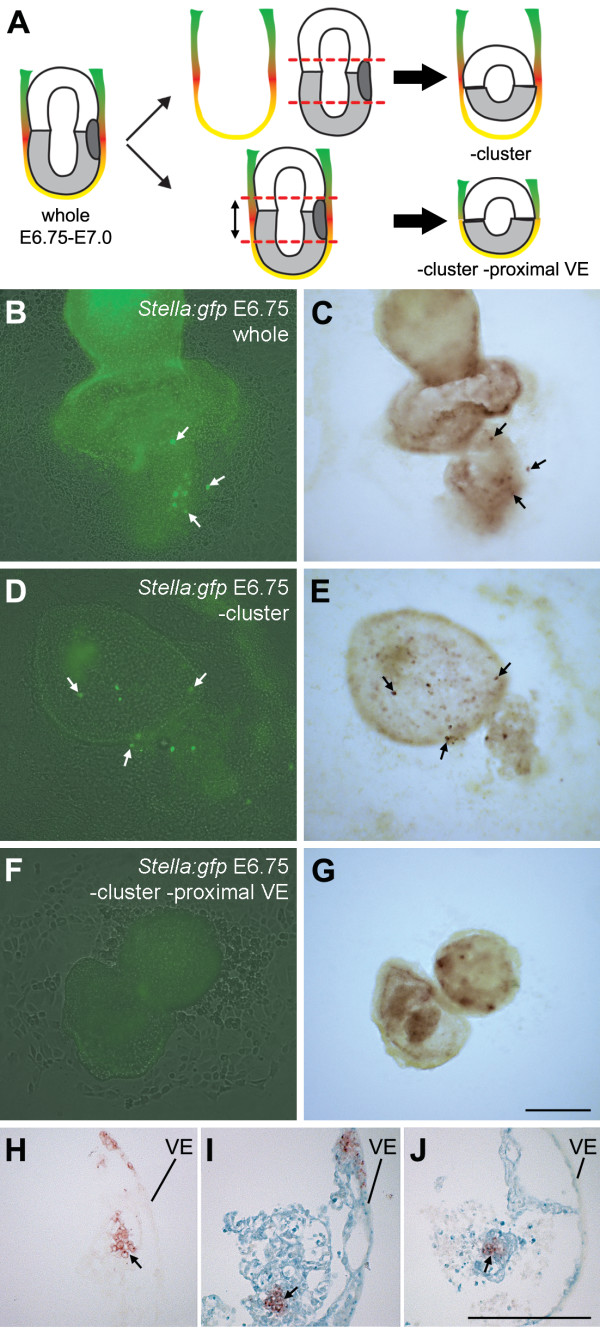
**Proximal VE and ExE are sufficient to induce PGCs in distal epiblast**. (A) Schematic of the experimental design. E6.75–E7.0 *Stella:gfp *embryos were isolated and either the VE was isolated whole (cup shaped), the proximal region containing the PGC precursor cluster surgically removed and the VE "filled" with the ExE and distal epiblast (-cluster); or the embryo was cut in three parts and the part containing the PGC precursor cluster discarded (-cluster -proximal VE). (B-G) Explants derived from E6.75–E7.0 whole (B, C), -cluster (D, E) and -cluster -proximal VE (F, G) immunostained to detect Stella(GFP) (B, D, F), followed by staining to detect alkaline phosphatase-activity (C, E, G). Arrows show same PGCs in the different panels. (H-J) Sagittal section showing double LacZ and alkaline phosphatase-activity staining in E6.75 wild type (H), *ROSA26:LacZ *(I) and -cluster (J) explants cultured for 3 days. In -cluster explants, the ExE and VE were from wild type embryos and the distal epiblast was from a *ROSA26:LacZ *embryos. Arrows show the PGC cluster in each explant; the VE is also indicated. Scale bar is 200 μm.

To directly test whether the VE and ExE can continuously recruit PGC precursors from the proximal epiblast cells between E6.25–E7.25, we investigated the capacity for the *de novo *formation of PGCs in E6.75–E7.0 *Stella:gfp *embryos from which the original cluster of PGC precursors had been removed. To do this, we either removed the entire VE (which has the form of a cup) and introduced inside a small piece of distal epiblast together with a small piece of ExE (-cluster) (Fig. 4A), or alternatively we removed the entire region of the embryo containing both the proximal epiblast and surrounding proximal VE, and brought the distal embryonic part in contact with the extraembryonic part (-cluster -proximal VE) (Fig. 4A). The reconstituted-embryos were analysed after 3 days of culture. In '-cluster -proximal VE' explants, we observed no induction of PGCs (n = 0/12), however in '-cluster' explants we were able to generate PGCs (n = 18/40, containing 1–38 PGCs) expressing both Stella(GFP) and TNAP (Fig. 4B–G). Together, the data clearly show that the combination of proximal VE and ExE is not only necessary, but also sufficient to induce PGCs in distal epiblast cells, which usually do not give rise to PGCs in the embryo.

To exclude the possibility that the induced PGCs resulted from contamination of proximal epiblast cells present in the ExE, we made reconstituted-embryos using *ROSA26:LacZ *distal epiblast and wild type VE and ExE. These reconstituted-embryos showed that the newly induced TNAP-positive PGCs are LacZ-positive, demonstrating that the PGCs indeed originated from the *ROSA26:LacZ *distal epiblast (Fig. 4H–J).

### PGC precursors still need a specific niche to undergo PGC specification

**Figure 5:**
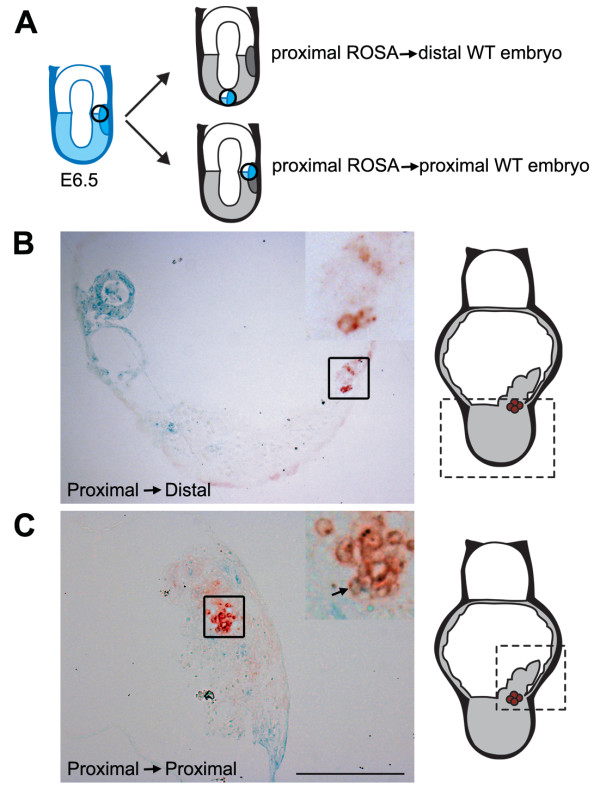
**At E6.5, PGC precursors can still change fate when placed heterotopically**. (A) Schematic of the experimental design. E6.5 *Blimp1:gfp ROSA26:LacZ *(ROSA) embryos were recovered and the proximal-posterior part containing Blimp1(GFP)-positive PGC precursors isolated and transplanted either distally of proximal-posteriorly into a E6.5 wild type (WT) embryo. The recombinates were cultured for 3 days and stained for LacZ and alkaline phosphatase-activity. (B, C) Sagittal section showing a recombinate explant in which the PGC precursor cluster were placed distally (B) and proximal-posteriorly (C). The inserts are a magnification of the region containing PGCs depicted in box. For orientation, a cartoon of the whole explant is included showing in the dashed box the region of the explant imaged. Scale bar is 200 μm.

In has been shown in a previous report that a group of E6.5 proximal posterior cells transplanted into a distal position in the embryo were unable to specify as PGCs [12]. However, the existence of the Blimp1-positive cluster of PGC precursors was then unknown. Therefore, it is possible that the group of proximal-posterior cells transplanted into the distal part of the embryo did not contain any such Blimp1-positive PGC-precursors. To unambiguously investigate whether Blimp1-expressing PGC precursors could specify to PGCs when placed in the distal epiblast, we transferred small groups of cells including Blimp1(GFP)-positive cells from *Blimp1:gfp ROSA26:LacZ *E6.5 embryos to the distal epiblast of E6.5 wild type embryos (n = 4) (Fig. 5A). These experiments demonstrated that the (LacZ-positive and initially Blimp1(GFP)-positive) cells could no longer give rise to TNAP-positive PGCs (Fig. 5B) after 3 days of culture. Instead, these cells were incorporated into morphologically more anterior structures faraway from the PGC region in the explant (Fig. 5B). By contrast, transplantation of groups of cells from a proximal-posterior (n = 4) location of E6.5 *Blimp1:gfp ROSA26:LacZ *embryos into a position close to the location of the PGC-precursor cluster in E6.5 wild type embryos (Fig. 5A), resulted in the formation of a LacZ-positive cluster in the posterior region of the embryo where a few double LacZ-positive and TNAP-positive PGCs were observed next to wild type PGCs (Fig. 5C). These results are in agreement with the previous report from [12] and together demonstrate that Blimp1-expressing PGC precursors still require local signals present exclusively in the proximal-posterior part of the embryo before they can undergo specification to PGCs.

## Discussion

### Regionalization of the embryo by ExE (and VE) regulates the formation of PGC precursors posteriorly

At E5.5, the anterior and posterior ExE exhibit already different characteristics important for the correct patterning (and migration) of the AVE and the subsequent patterning (and migration) of the epiblast [13,14]. At E5.75, the ExE may no longer influence the migration of the AVE, but it still plays a crucial role in regionalizing the epiblast, allowing gastrulation to take place. Indeed, *Elf5 *mutants, which contain no ExE, showed defects in embryonic patterning and failure to undergo gastrulation, despite the fact that the AVE is well specified [15]. In agreement, our data also suggests that ExE plays a critical role at E5.75–E6.0 in regionalizing the epiblast with respect to where the PGC precursors can emerge.

The anterior ExE (and AVE) does not support development of PGCs and may even inhibit the formation of Blimp1-positive cells. However, it is well known that cells lying throughout the perimeter of the proximal epiblast at E6.0 can potentially give rise 40 hour later to PGCs in the posterior part of the embryo [3]. These proximal cells move to the posterior part of the embryo due to the characteristic morphogenetic movements occurring during gastrulation. This directional movement allows some of these cells either to avoid signals from the anterior part of the embryo or to receive the necessary signals in the posterior part to become PGC progenitors.

### Induction of PGC precursors (by the VE and ExE) occurs gradually between E6.25–E7.25

The VE at E5.75 shows asymmetric expression of several secreted factors, including Cerl and Lefty1 in the AVE, important for the formation of the anteroposterior axis [16,17]. By contrast, there is little evidence for a direct role of the posterior part of the VE in embryonic patterning.

Our data suggests that the proximal-posterior VE plays a role in the generation of the full population of PGC precursors in the posterior part of the embryo. The role of the proximal-posterior VE could be that of promoting proliferation of the initial pool of PGC progenitors and therefore removing the VE would block cell division of the PGC progenitors, resulting in a smaller PGC population. Alternatively, as the VE is required for general cell survival in culture, we cannot exclude the possibility that normal numbers of PGC precursors were formed but died and could not be traced. However, we favor the possibility that when the VE is removed no more PGC precursors emerge but the ones allocated are able to undergo specification, although due to limitation of the culture system used some degenerate. Therefore, we propose that the proximal-posterior VE (together with the ExE) is actively recruiting additional PGC precursors from epiblast cells between E6.25–E7.25. This model also explains our observations that an E6.5 embryo is able to generate a PGC cluster *de novo*, when its original cluster is removed and that E6.5 distal epiblast cells when placed proximally (near a PGC precursor existing cluster) are still able to adopt a PGC fate (data not shown).

### Are neighbouring somatic cells providing PGC precursors with the molecular niche needed for specification?

The results presented show that cross talk between the posterior epiblast, the proximal VE and ExE is needed for the emergence of Blimp1-positive PGC precursors. In addition, we also show that Blimp1-positive PGC precursors do not autonomously undergo specification to PGCs. It is likely that extra inductive signals present locally trigger the specification of Blimp1-positive PGC precursors. The proposed extra inductive signals most probably originate from the extraembryonic mesoderm (ExM) surrounding the PGC progenitor cluster and not the VE and ExE. In contrast to our ideas, Yoshimizu and colleagues (2001) observed that E6.75 dissociated epiblast cells could give rise to PGCs [6], however they cultured those epiblast cells in the presence of feeder cells, which could be providing the necessary signals for specification.

One of the candidate secreted molecules exclusively expressed by the ExM, and not by PGCs, at E7.5 is BMP4 [18]. In addition, a necessary event during PGC specification is the downregulation of E-cadherin [19], followed by the acquisition of a motile phenotype. Both FGF and WNT signalling pathways are known to regulate gastrulation and can downregulate E-cadherin [20-24]. Further research will demonstrate whether the BMP, WNT or FGF signalling pathways are directly involved in PGC specification.

## Conclusion

The recent evidence for a population of PGCs progenitors in the mouse embryo has compelled us to re-evaluate the role of the extraembryonic tissues in the formation of PGCs. Using transgenic mouse models in combination with an explant-assay, we determined that the extraembryonic tissues, VE and ExE, as part of their patterning role during gastrulation, are also responsible for the correct induction of PGC progenitors. However, the VE and ExE do not seem to be directly involved in the specification to PGCs as expected. We support the idea that not only the VE, but also the ExE is regionalized and that it is the cross talk in the proximal-posterior part of the embryo that allows the development of PGC progenitors at E6.25–E7.25. To date, little importance has been given to the posterior VE, in contrast to the AVE. The ExE and VE seem to play different roles, but both important for the normal development of PGC progenitors. Finally, being a PGC progenitor is not sufficient to become a specified PGC and signals probably secreted from the ExM should prove to induce PGC specification.

## Methods

### Animals, isolation of embryos and culture of explants

All experiments involving animals were approved by the Home Office, UK. Wild type mice and transgenic *Blimp1:gfp*, *Stella:gfp*, *ΔPEOct4:gfp *and *Cerl:gfp *mice were on a mixed C57BL/6 and CBA background, *ROSA26:LacZ *mice were C57BL/6. Noon of the day of vaginal plug was designated E0.5. Embryos were isolated at E5.75–E6.0 and E6.75–E7.0 in cold Dulbecco's minimal medium (DMEM, Invitrogen) supplemented with 7.5% fetal calf serum (FCS) and 10 mM HEPES. *Blimp1:gfp *embryos were selected from non-transgenic littermates under an Olympus IX71 inverted microscope (London, UK). If necessary, the VE was removed as described previously [5]. Embryos were cut (1) longitudinally through the long L-R axis; (2) transversely at several heights in the presence of absence of the VE; or (3) parts of one or several different embryos were recombined using tungsten needles. Thereafter, they were cultured in DMEM+15% FCS at 37°C and 5% CO_2 _on air either on fibronectin (20 μg/ml) coated glass coverslips in 4 well culture plates (NUNC) or in suspension in 3 cm bacteriologic dishes. The two methods were used to obtain explants attached to a coverslip or to maintain the explants in suspension, respectively. The two methods gave similar results in terms of PGC development. Explants derived from E5.75–E6.0 embryos were cultured for maximal 4 days and explants derived from E6.75–E7.0 embryos for maximal 3 days. Fresh embryos and explants were imaged using an Olympus IX71 (London, UK) inverted microscope.

### Immunofluorescence

Immunofluorescence staining of explants on coverslips was performed essentially as described [5]. The primary antibodies used were rat anti-GFP (1:500, Nacalai Tesque) and rabbit anti-PGC7/Stella (1:2000); and the secondary antibodies used were from Molecular Probes. Explants were imaged using an Olympus IX71 (London, UK) inverted microscope or a Bio-Rad Radiance 2000 confocal microscope (CA, USA).

### Cytological stainings and histology

Some of the explants (grown in suspension or on coverslips) were used for detection of alkaline phosphatase-activity. Briefly, the explants were washed three times in PBS, kept 1 hour in 70% EtOH at 4°C, washed three times with distilled water and treated with an aqueous solution containing 1% veronal (Sigma), 0.12% MgCl_2_, 0.02 mg/ml α-naphtyl phosphate (Sigma) and 1 mg/ml Fast Red TR (Sigma).

Recombinates of wild type and *ROSA26:LacZ *embryos were fixed in 4% paraformaldehyde in PBS, stained for LacZ using standard procedures, embedded in Technovit 8100 (Heraeus Kulzer), sectioned (6 μm) and stained for alkaline phosphatase-activity using ASMX/Fast Red TR (Sigma) following manufacturer's instructions. Explants were imaged using an Olympus IX71 (London, UK) inverted microscope.

## List of abbreviations

AVE: anterior visceral endoderm; BMP: bone morphogenetic protein; E: embryonic day; ExE: extraembryonic ectoderm; GFP: green fluorescent protein; PGCs: primordial germ cells; TNAP: tissue non-specific alkaline phosphatase; VE: visceral endoderm

## Authors' contributions

SMCSL isolated, manipulated and cultured all embryos and recombinates; performed immunofluorescence, histological and cytological stainings; quantified and imaged embryos; participated in the design of the study and prepared the manuscript. KH imaged PGCs in confocal; participated in the design of the study and revised the manuscript. MAS participated in the design of the study and revised the manuscript.
